# Review of "RF/Microwave Interaction with Biological Tissues" by André Vander Vorst, Arye Rosen, and Youji Kotsuka

**DOI:** 10.1186/1475-925X-5-50

**Published:** 2006-09-20

**Authors:** Isaac Chang

**Affiliations:** 1 Office of Science and Engineering Laboratories, Center for Devices and Radiological Health, U.S. Food and Drug Administration, Rockville, MD 20852, USA

In today's world, the interaction of radiofrequency (RF) and microwave fields has garnered much attention and is increasing in importance in both the public health and medical device communities. The subject is complex and difficult to master as it draws on several sub-areas of study. Until recently, the only means for students and researchers to familiarize themselves with the intricacies of RF/Microwave tissue interactions was to read the many manuscripts and critical reviews that have been published in the last 60–70 years. The literature is both voluminous and detailed with few guides to help facilitate introduction to new researchers and engineering students. This book presents an overview of important background topics needed for an introduction to RF/Microwave interactions with body tissues (100 kHz- 10 GHz).

The book is organized into six chapters. Chapter 1: Fundamentals in Electromagnetics provides a summary of the basic electromagnetics as it applies to biological tissues. Unlike traditional texts on electromagnetics, this chapter presents the major theoretical points as "observations." The chapter mainly focuses on the relationship between field quantities and the description of static/dynamic movement of charge. The chapter also focuses on the penetration of fields in biological tissues, the description and plotting techniques for plotting complex admittance (i.e. Cole-Cole), the effects of fields in the near field, blackbody radiation, and microwave measurement techniques.

Chapter 2: RF/Microwave Interaction Mechanisms in Biological Materials is a collection of sub-topics that largely describe the mechanisms that occur when fields interact with tissues. Sub-topics such as polarization and relaxation are introduced. Descriptions of conductivity and permittivity, and typical values measured in various tissues are presented. The chapter ends with a description of the fundamentals of thermodynamics and a discussion of energy and entropy.

Chapter 3: Biological Effects presents a description of the methods and metrics used to describe exposure of RF/Microwave energy to biological tissues. The first part of the chapter focuses on a description of the specific absorption rate (SAR) and thermal distribution formulations. It introduces the reader to SAR limits for different biological tissues. The chapter then focuses on the published effects of RF fields on the nervous system, the brain and spinal cord, the blood-brain barrier, and cell and membranes. The chapter finishes by presenting experimental and computational methods for assessing SAR in tissues.

Chapter 4: Thermal Therapy presents an extensive description of the different modes of RF heating. It focuses mainly on dielectric and inductive heating. Both the description of dielectric heating (from electric fields) and inductive heating (from magnetic fields) apply to relatively high frequency environments. The chapter does not focus on conductive heating, which also occurs and is the primary mode of thermal heating for RF medical devices (480–550 kHz). [1] The chapter ends with a description of thermometry methods and measurement using thermistor and thermocouple sensors.

Chapter 5: EM-Wave Absorbers Protecting the Biological and Medical Environment presents an extensive discussion on the materials used for electromagnetic wave absorbers. Rather than focusing on biological tissues, the authors chose to focus on the technical and theoretical design of EM wave and ferrite absorbers. While the authors make a compelling argument as to its utility, the subject is distinctly different than, and is covered on a level of detail that far exceeds, the material in any other chapter of the book.

The last chapter, Chapter 6: RF/Microwave Delivery Systems for Therapeutic Applications, covers some of the fundamental features of general RF/microwave delivery devices (i.e., antennas and waveguides). The chapter discusses some of the medical applications for RF and microwave energy. The actual discussion of RF topics is extremely limited, compared to the extensive discussion on microwave issues. The chapter ends with a description of new research areas that may impact the development of future delivery systems.

Overall, the book is useful as an introductory book on the subject of RF/Microwave interaction with biological fields. It serves as a handy guide to organizing the various major sub-topics. Perhaps, the most useful aspect of the book is its extensive chapter reference citations, which list all of the classic texts for further study. In reviewing the book, I found several limitations. First, with regard to the material covered, the book overemphasizes the microwave interaction topics over its low frequency RF counterparts. Several important concepts, such as the different definitions for tissue impedance (at high and low frequency) were not presented. The topic of quasi-static approximations was also notably missing. Given the prevalence of computational modeling today, a critique of approximation schemes for solving low frequency RF problems appears to be an important topic. Another oddity was the organization of Chapter 2 and 3, which barely delineated differences in the terms "interaction" and "effects."

Despite its limitations, the book does a noble job introducing a difficult and diverse topic. It provides a survey for a broad range of material and provides a few problems for biomedical engineering students. The authors' intent to use the book to develop new interest and new biomedical courses can be realized by using this book as a supplemental source to classic electromagentics texts.

DISCLAIMER: The opinions and/or conclusion expressed are solely those of the authors and in no way imply a policy or position of the Food and Drug Administration.
